# Study on *x*LiVPO_4_F·*y*Li_3_V_2_(PO_4_)_3_/C Composite for High-Performance Cathode Material for Lithium-Ion Batteries

**DOI:** 10.3389/fchem.2020.00361

**Published:** 2020-05-08

**Authors:** Shengkui Zhong, Xiaoping Zhang, Jiequn Liu, Yulei Sui

**Affiliations:** ^1^School of Marine Science and Technology, Hainan Tropical Ocean University, Sanya, China; ^2^School of Iron and Steel, Soochow University, Suzhou, China

**Keywords:** lithium-ion batteries, cathode material, LiVPO_4_F, Li_3_V_2_(PO_4_)_3_, electrochemical performance

## Abstract

Cathode materials made of *x*LiVPO_4_F·*y*Li_3_V_2_(PO_4_)_3_/C (*x:y* = 1:0, 2:1, 0:1) are synthesized via a feasible sol-gel method for high-performance lithium-ion batteries. The structures, morphology, and electrochemical properties of the composites are thoroughly investigated. The results show that LiVPO_4_F/C, Li_3_V_2_(PO_4_)_3_/C, and 2LiVPO_4_F·Li_3_V_2_(PO_4_)_3_/C can be synthesized under 750°C without the formation of impurities. Meanwhile, the unique morphology of the 2LiVPO_4_F·Li_3_V_2_(PO_4_)_3_/C composite, which is porous, with nanoflakes adhering to the surface, is revealed. This composite integrates the advantages of LiVPO_4_F and Li_3_V_2_(PO_4_)_3_. There are four discharge plateaus near 4.2, 4.1, 3.7, and 3.6 V, and the cathode material delivers high capacities of 143.4, 141.6, 133.2, 124.1, and 117.6 mAh g^−1^ at rates of 0.1, 0.2, 0.5, 1, and 2 C, respectively. More importantly, the discharge capacity can be almost fully recovered when the discharge rate returns to 0.1 C. The study is highly promising for the development of cathode material for LIBs.

## Introduction

With the increasing demand for renewable energy resources, lithium-ion batteries (LIBs) have attracted tremendous attention in recent years (Uddin et al., 2017; Wu et al., 2019; Zheng et al., 2019). As one of the most important parts of LIBs, cathode materials play a critical role in their electrochemical performance (Goodenough and Park, 2013; Nitta et al., 2015; Zheng et al., 2017; Sui et al., 2019a). Recently, various materials have been explored for LIB cathodes, such as olivine-structured materials (LiMPO_4_, M = Fe, Mn, Co, and Ni) (Li Y. et al., 2019; Vásquez and Calderón, 2019; Wu et al., 2020), layered oxide materials (LiCoO_2_, LiMnO_2_, LiNiO_2_, etc.) (El-Bana et al., 2017; Zhao et al., 2017; Lin et al., 2020), spinel-structured materials (LiMn_2_O_4_, LiCo_2_O_4_, etc.) (Hariprasad et al., 2016; Abou-Rjeily et al., 2019), tavorite-structured materials (LiFeSO_4_F, LiVPO_4_F, etc.) (Kim and Kang, 2017; Hu et al., 2019), and NASICON-structured materials [Li_3_V_2_(PO_4_)_3_, Li_3_Fe_2_(PO_4_)_3_, etc.] (Karami and Taala, 2011; Zheng et al., 2016; Jiang, 2018).

Among these materials, LiVPO_4_F and Li_3_V_2_(PO_4_)_3_ are especially promising and have drawn attention in recent years. LiVPO_4_F, which was synthesized by Barker et al. (2003) in 2003 for the first time, is attracting attention due to its high theoretical energy density (655 Wh kg^−1^) and high working voltage platform (4.2 V vs. Li/Li^+^) (Gover et al., 2006). The synergistic effect between F^−^ and ${\text{PO}}_{4}^{3-}$ within the material could enhance the electronegativity and induction property of LiVPO_4_F, achieving improved electrochemical performance (Huang et al., 2009). For example, Sui et al. (2019b) have prepared spherical LiVPO_4_F/C by spray drying, and the initial discharge capacity of the synthesized LiVPO_4_F/C can be as high as 137.9 mAh g^−1^ at 0.1 C, and discharge capacity remains at 91.4% of the initial capacity after 50 cycles. However, the Li^+^ diffusion coefficient in LiVPO_4_F is low, which restricts its application.

Li_3_V_2_(PO_4_)_3_, a typical NASICON-structured material, allows the insertion and/or extraction of lithium ions from various pathways, and exhibits an outstanding Li^+^ diffusion coefficient (10^−9^-10^−10^ cm^2^ s^−1^) (Li R. et al., 2019). In addition, Li_3_V_2_(PO_4_)_3_ displays a high average operation voltage (4.0 V vs. Li^+^/Li) and excellent theoretical capacity (197 mAh·g^−1^) (Zhang et al., 2019). Therefore, it is expected that the combination of Li_3_V_2_(PO_4_)_3_ with LiVPO_4_F may enhance the ionic conductivity of cathode material and improve electrochemical performance.

Herein, we synthesized *x*LiVPO_4_F·*y*Li_3_V_2_(PO_4_)_3_/C cathode material for LIBs via a feasible sol-gel method. Such a design is demonstrated to combine the advantages of LiVPO_4_F and Li_3_V_2_(PO_4_)_3_, and it can effectively enhance electronic/ionic conductivity and structural stability. The optimal *x*LiVPO_4_F·*y*Li_3_V_2_(PO_4_)_3_/C shows a high initial discharge capacity, outstanding rate capability, and proper cycling performance, illustrating that it is a promising cathode material for LIBs.

## Experimental Method

Synthesis of LiVPO_4_F/C: firstly, stoichiometric amounts of H_3_PO_4_, V_2_O_5_, and citric acid were added into deionized water under stirring, and then ammonia was added into the solution. After stirring at 80°C for 6 h and drying overnight in an oven, the precursor of VPO_4_ was obtained. The obtained VPO_4_ was pretreated at 300°C for 4 h under the protection of Ar gas and heated at 750°C for 8 h to prepare VPO_4_/C. Finally, stoichiometric amounts of the VPO_4_-based precursor and LiF were mixed and calcined from 690 to 770°C for 1 h under a flowing Ar atmosphere to obtain LiVPO_4_F/C. All chemical reagents used in this work were analytical grade.

Synthesis of Li_3_V_2_(PO_4_)_3_/C: stoichiometric amounts of the prepared VPO_4_ precursor and Li_3_PO_4_ were mixed and calcined from 650 to 800°C for 16 h under a flowing Ar atmosphere to obtain Li_3_V_2_(PO_4_)_3_/C.

Synthesis of 2LiVPO_4_F·Li_3_V_2_(PO_4_)_3_/C: Stoichiometric amounts of the prepared VPO_4_/C, LiF, and Li_3_PO_4_ were mixed and calcined at 750°C for 1 h under a flowing Ar atmosphere to obtain 2LiVPO_4_F·Li_3_V_2_(PO_4_)_3_/C samples.

Crystallographic information was studied by powder X-ray diffractometer (XRD, Rigaku, Ultima VI). The morphology was observed with a Hitachi SU5000 scanning electron microscope (SEM). The electrodes for electrochemical tests were fabricated with 80 wt.% active material and 20 wt.% additives (PVDF/acetylene black, 1:1). CR2025 button cells were assembled in an Ar-filled glove box. The galvanostatic charge/discharge and the cyclic voltammetry (CV) were characterized with a LAND battery test system and a CHI660D electrochemical workstation, respectively.

## Results and Discussion

**Figure 1:**
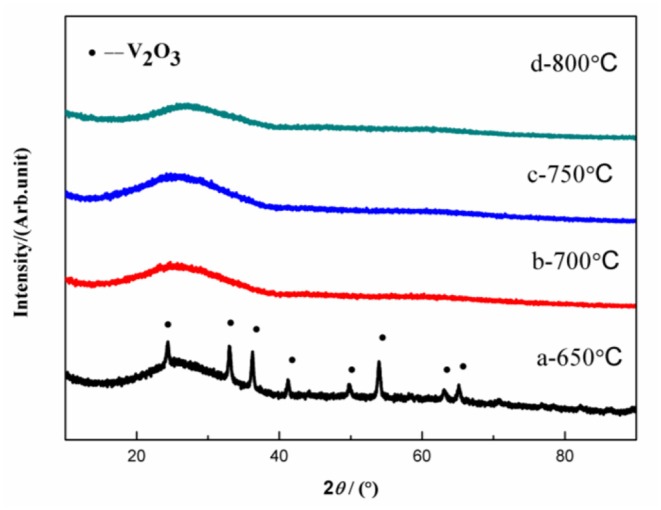
XRD patterns of VPO_4_/C precursors synthesized at different temperatures.

Figure 1 shows the XRD results of the VPO_4_/C precursors synthesized at different temperatures. Obvious peaks indexed to V_2_O_3_ are observed in the precursor calcined at 650°C. However, these peaks disappear when the calcination temperature is elevated above 700°C. Hence, the precursors synthesized at 700, 750, and 800°C can be confirmed to be amorphous VPO_4_/C. Meanwhile, the absence of peaks belong to crystalline carbon suggests the amorphous nature of the carbon coating layer.

**Figure 2:**
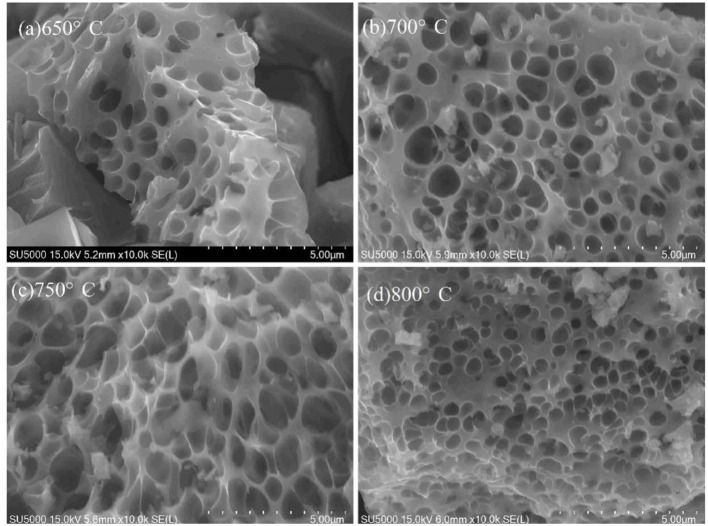
SEM images of the VPO_4_/C precursors synthesized at different temperatures. **(a)** 650°C; **(b)** 700°C; **(c)** 750°C; **(d)** 800°C.

The morphology of VPO_4_/C precursors synthesized at various temperatures was also investigated, as shown in Figure 2. Figure 2a suggests that the reactants cannot be completely converted to products at 650°C. Moreover, the pores on the samples are uneven. As the temperature rises to 700°C, the product has a more uniform pore diameter. However, many small particles adhere to the surface of the sample. At 750°C, the pores become most uniform, and the small particles disappear. A serious aggregation occurs when the temperature further increases to 800°C. Therefore, the optimal calcination temperature for VPO_4_/C precursor is 750°C, which is chosen for the synthesis of *x*LiVPO_4_F·*y*Li_3_V_2_(PO_4_)_3_/C (*x:y* = 1:0, 2:1, 0:1).

**Figure 3:**
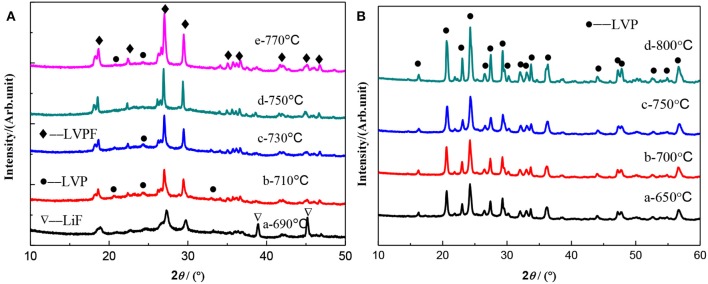
XRD patterns of LiVPO_4_F/C **(A)** and Li_3_V_2_(PO_4_)_3_/C **(B)** synthesized at different temperatures.

XRD patterns of LiVPO_4_F/C (Figure 3A) and Li_3_V_2_(PO_4_)_3_/C (Figure 3B) synthesized at different temperatures are shown in Figure 3. As can be seen, the diffraction peaks are stronger and the half-peak width narrower at the higher calcination temperature, indicating that elevated temperature facilitates the crystallization of composites. It can be seen in Figure 3A that the characteristic peaks of Li_3_V_2_(PO_4_)_3_ and LiF become weaker as the temperature increases and that they completely disappear at 750°C. However, the impurity of Li_3_V_2_(PO_4_)_3_ reappears when the temperature reaches 770°C, suggesting that an excessive temperature has a detrimental effect on the formation of LiVPO_4_F. On the contrary, only pure Li_3_V_2_(PO_4_)_3_ is observed in the temperature range from 650 to 800°C in Figure 3B.

**Figure 4:**
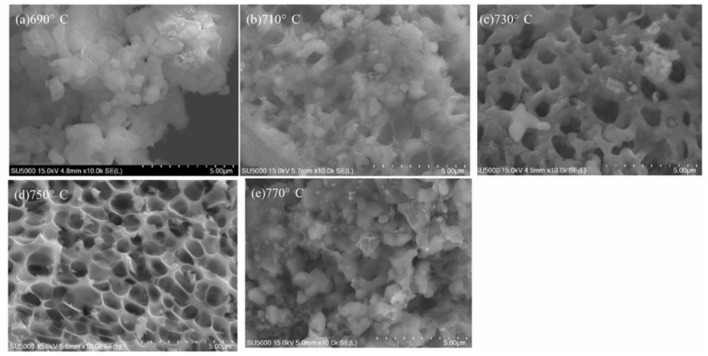
SEM images of LiVPO_4_F/C synthesized at different temperatures. **(a)** 690°C; **(b)** 710°C; **(c)** 730°C; **(d)** 750°C; **(e)** 770°C.

**Figure 5:**
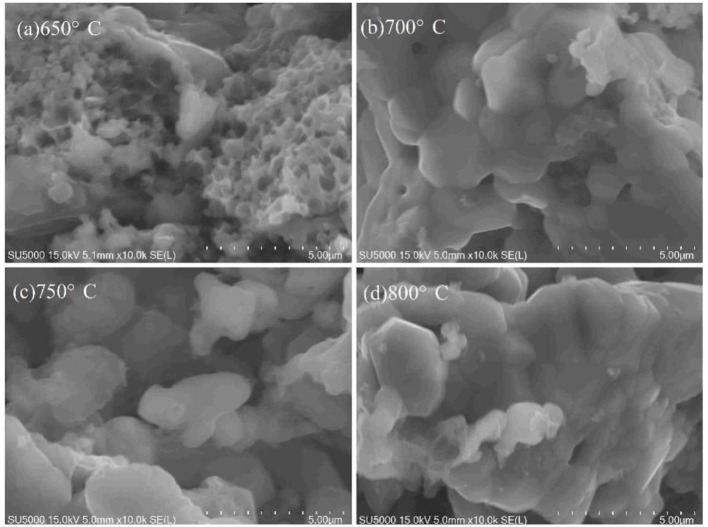
SEM images of Li_3_V_2_(PO_4_)_3_/C synthesized at different temperatures. **(a)** 650°C; **(b)** 700°C; **(c)** 750°C; **(d)** 800°C.

Figure 4 shows SEM images of LiVPO_4_F/C synthesized at different temperatures. Obviously, the samples obtained at 730 and 750°C possess a porous morphology, and the latter has a more uniform pore diameter. In contrast, those calcined at lower temperatures (690 and 710°C) are not porous, since there are lots of unreacted LiF particles left on the surface of the samples. At 770°C, all pores disappear due to aggregation. Figure 5 shows SEM images of Li_3_V_2_(PO_4_)_3_/C synthesized at different temperatures. Although the XRD results (Figure 3b) do not show a significant difference between these samples calcined at different temperatures, they can be clearly distinguished from each other in the SEM images, indicating that the porous VPO_4_/C and Li_3_PO_4_ start to react at around 650°C. With an increase in temperature, the particles coarsen since the high temperature can accelerate the growth rate of particles. When calcined at 750°C, the particles display irregular shapes and are uniformly dispersed on the carbon network. As the temperature reaches 800°C, the particles agglomerate seriously. Hence, the optimal calcination temperature for synthesis of LiVPO_4_F/C and Li_3_V_2_(PO_4_)_3_/C is 750°C.

**Figure 6:**
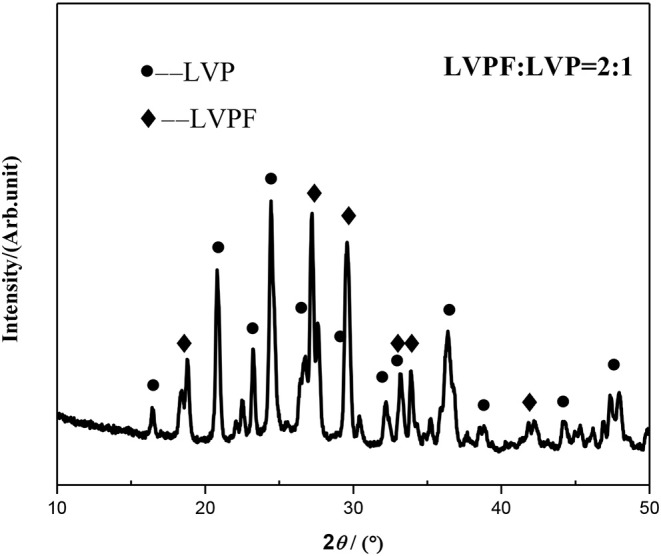
XRD pattern of 2LiVPO_4_F·Li_3_V_2_(PO_4_)_3_/C.

Based on the above analysis, 2LiVPO_4_F·Li_3_V_2_(PO_4_)_3_/C was chosen to be synthesized at 750°C. The XRD pattern of the as-prepared 2LiVPO_4_F·Li_3_V_2_(PO_4_)_3_/C is shown in Figure 6. It can be seen that all diffraction peaks can be indexed to LiVPO_4_F and Li_3_V_2_(PO_4_)_3_, and no impurity is detected. Moreover, characteristic peaks of crystalline carbon cannot be spotted, suggesting that carbon is amorphous in the composite.

**Figure 7:**
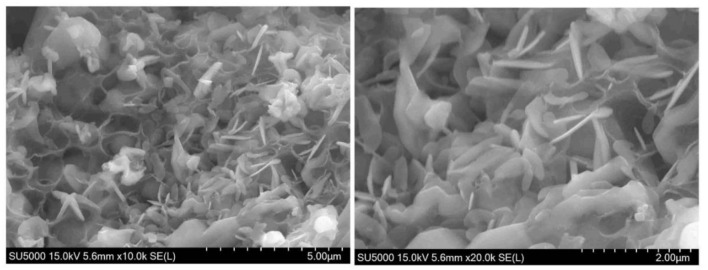
SEM images of the as-prepared 2LiVPO_4_F·Li_3_V_2_(PO_4_)_3_/C.

Figure 7 displays SEM images of the as-prepared 2LiVPO_4_F·Li_3_V_2_(PO_4_)_3_/C. The composite is composed of porous particles with a pore diameter of 0.5–2 μm and nanosheets with a thickness of 200–500 nm. Furthermore, the amorphous carbon can be clearly observed. This uniform morphology of this material could facilitate ion transportation.

**Figure 8:**
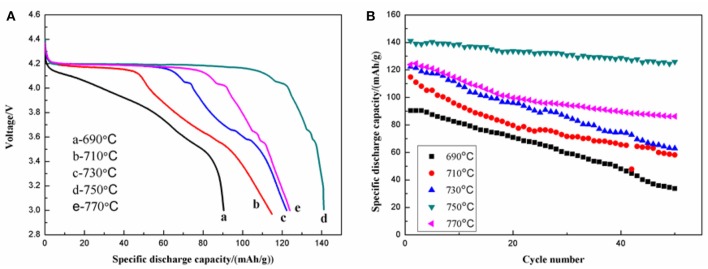
Initial discharge curves **(A)** and cycling performance **(B)** of LiVPO_4_F/C synthesized at different temperatures.

The electrochemical performance of the as-prepared materials was tested using galvanostatic charge-discharge in the voltage range of 3.0–4.5 V. The initial discharge curves (Figure 8A) and the cycling performances (Figure 8B) of the LiVPO_4_F/C samples were revealed. The one calcined at 750°C delivers the highest initial discharge capacity of 141.1 mAh g^−1^ at 0.1 C. After 50 cycles, the remaining capacity can be as high as 125.9 mAh g^−1^, with a capacity retention of 89.22%. The samples calcined at 690, 710, 730, and 770°C display capacities of 90.4, 114.8, 122.3, and 123.9 mAh g^−1^ at 0.1 C, respectively. The corresponding capacity retention rates after 50 cycles are 37.30, 50.69, 51.43, and 69.57%. The samples calcined in the ranges of 710–770°C show the characteristic discharge plateaus of LiVPO_4_F, as well as the characteristic discharge plateaus of Li_3_V_2_(PO_4_)_3_ around 4.10, 3.68, and 3.58 V. For the sample calcined at 750°C, no diffraction peaks of Li_3_V_2_(PO_4_)_3_ are detected in the XRD pattern, but it indeed shows the characteristic discharge plateaus. This is due to the very tiny amount of Li_3_V_2_(PO_4_)_3_ in this sample. Moreover, this sample has a good three-dimensional porous structure, which provides pathways for Li-ion transportation, consequently enhancing the cycling performance.

**Figure 9:**
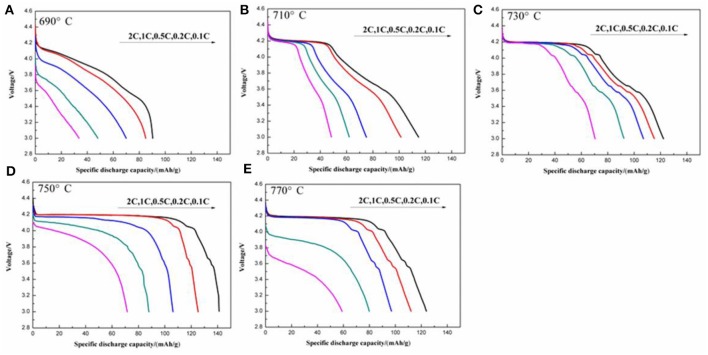
Initial discharge curves at different discharge rates of the LiVPO_4_F/C synthesized at different temperatures. **(A)** 690°C; **(B)** 710°C; **(C)** 730°C; **(D)** 750°C; **(E)** 770°C.

The initial discharge curves at various discharge rates of the LiVPO_4_F/C samples synthesized at different temperatures are illustrated in Figure 9. The one calcined at 750°C shows a better rate capability than the others. For example, the initial discharge capacities at 0.1, 0.2, 0.5, 1, and 2 C are 141.1, 125.2, 106.3, 87.9, and 71.5 mAh g^−1^, respectively. The discharge plateau around 4.17 V becomes lower and shorter with an increase in discharge rate, indicating that the high current density enlarges the resistance of the electrochemical reactions.

**Figure 10:**
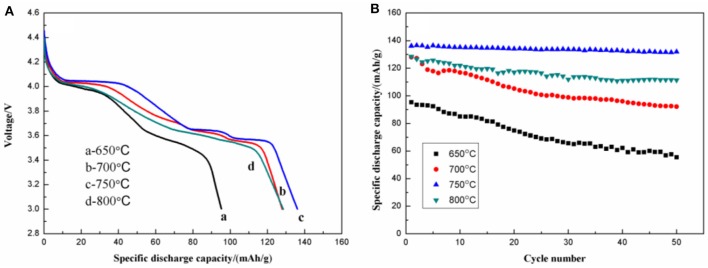
Initial discharge curves **(A)** and cycling performance **(B)** of the Li_3_V_2_(PO_4_)_3_/C synthesized at different temperatures.

Figure 10 shows the initial discharge curves (Figure 10A) and the cycling performance (Figure 10B) of Li_3_V_2_(PO_4_)_3_/C. The samples calcined at 650, 700, 750, and 800°C show initial discharge capacities of 95.3, 128.0, 136.1, and 128.5 mAh g^−1^, respectively. After 50 cycles, the remaining capacities are 55.5, 92.2, 131.9, and 111.4 mAh g^−1^, with corresponding capacity retentions of 58.2, 72.0, 96.9, and 86.7%. Compared with the samples calcined at 650, 700, and 800°C, the one calcined at 750°C shows more stable discharge plateaus at 4.07, 3.68, and 3.58 V, suggesting suppressed polarization and a more stable structure.

**Figure 11:**
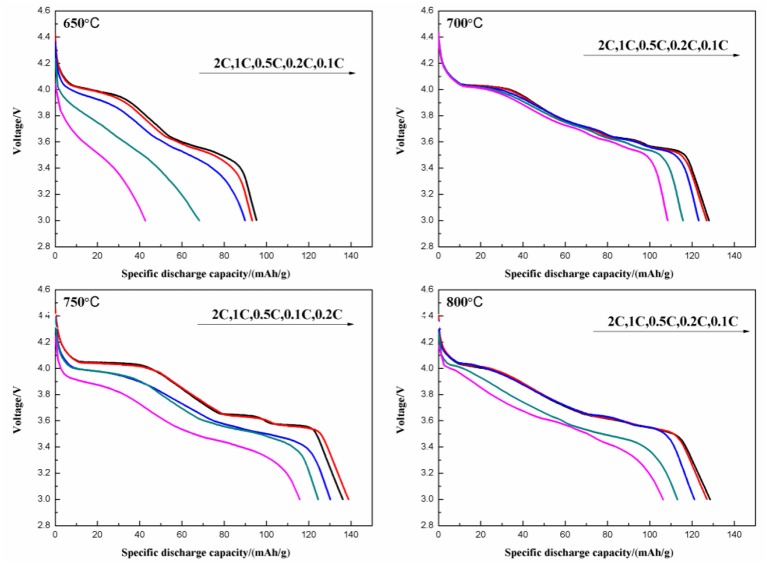
Initial discharge curves at different discharge rates of Li_3_V_2_(PO_4_)_3_/C synthesized at different temperatures.

Figure 11 shows the initial discharge curves at various discharge rates of the Li_3_V_2_(PO_4_)_3_/C synthesized at different temperatures. The one calcined at 750°C delivers the highest discharge capacity. When tested under rates of 0.1, 0.2, 0.5, 1, and 2 C, it shows discharge capacities of 136.1, 138.8, 130.2, 124.4, and 115.7 mAh g^−1^, respectively. Moreover, the discharge plateaus are stable even under higher current density.

**Figure 12:**
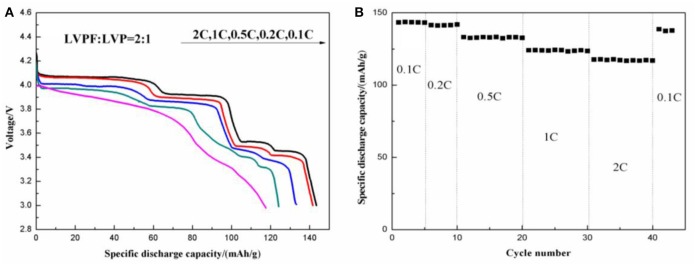
Initial discharge curves at different discharge rates **(A)** and rate capability **(B)** of the as-prepared 2LiVPO_4_F·Li_3_V_2_(PO_4_)_3_/C.

Figure 12 shows the electrochemical properties of 2LiVPO_4_F·Li_3_V_2_(PO_4_)_3_/C. The initial discharge curves at different discharge rates (Figure 12A) show that this composite delivers a significantly improved electrochemical performance in comparison with LiVPO_4_F/C and Li_3_V_2_(PO_4_)_3_/C. For instance, 2LiVPO_4_F·Li_3_V_2_(PO_4_)_3_/C has an initial discharge capacity of 143.1 mAh g^−1^ at 0.1 C (1 C = 156 mA g^−1^), which is higher than that of the other materials. With an increase in current density, the capacity fading is minor. When tested at 0.2, 0.5, 1, and 2 C, the discharge capacities are 141.6, 133.2, 124.1, and 117.6 mAh g^−1^, respectively. The discharge curves show four plateaus. The one around 4.2 V is attributed to the discharge of LiVPO_4_F, and the others around 4.1, 3.7, and 3.6 V are due to the discharge of Li_3_V_2_(PO_4_)_3_. Figure 12B shows the rate capability of the composite. After being successively tested at 0.1 and 0.2 C for 5 cycles and 0.5, 1, and 2 C for 10 cycles (40 cycles in total), the discharge capacity can be almost fully recovered when the discharge rate returns to 0.1 C.

**Figure 13:**
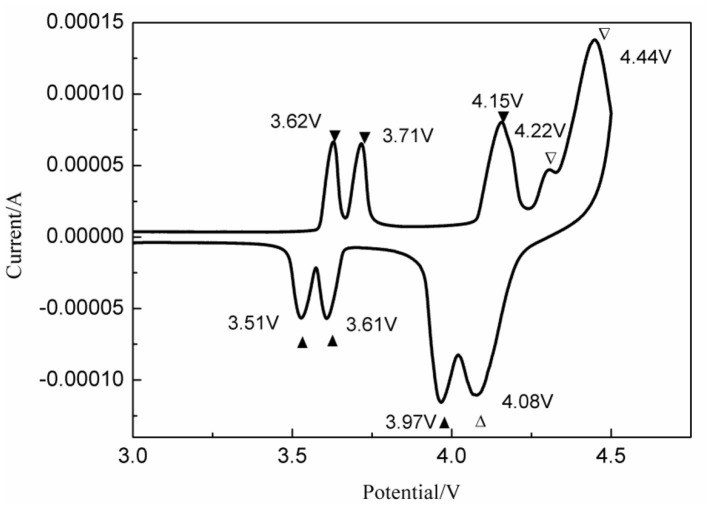
CV curves of the as-prepared 2LiVPO_4_F·Li_3_V_2_(PO_4_)_3_/C.

In order to study the lithiation/delithiation processes of 2LiVPO_4_F·Li_3_V_2_(PO_4_)_3_/C, a CV test was implemented; the results are shown in Figure 13. The anodic peaks at 4.22 and 4.44 V and the cathodic peak at 4.08 V correspond to the reversible redox of V^3+^/V^4+^ in LiVPO_4_F. The potential difference is 0.36 V. Moreover, the anodic peaks at 3.62, 3.71, and 4.15 V and the corresponding cathodic ones at 3.51, 3.61, and 3.97 V are due to the multi-step Li-ion extraction/insertion in Li_3_V_2_(PO_4_)_3_. The potential differences are 0.11, 0.10, and 0.18 V. Apparently, the polarization in Li_3_V_2_(PO_4_)_3_ is much smaller than that in LiVPO_4_F. Hence, a moderate amount of Li_3_V_2_(PO_4_)_3_ could suppress the polarization in the *x*LiVPO_4_F·*y*Li_3_V_2_(PO_4_)_3_/C composite, consequently enhancing the electrochemical performance.

## Conclusions

In summary, *x*LiVPO_4_F·*y*Li_3_V_2_(PO_4_)_3_/C (*x:y* = 1:0, 2:1, 0:1) composites were successfully synthesized via a feasible sol-gel method. Our results indicate that the composites are well-crystallized and composed of porous particles. 2LiVPO_4_F·Li_3_V_2_(PO_4_)_3_/C outperforms LiVPO_4_F/C and Li_3_V_2_(PO_4_)_3_/C due to the synergy between the fluorophosphate and phosphate. This study presents a facile approach in synthesizing *x*LiVPO_4_F·*y*Li_3_V_2_(PO_4_)_3_/C for high-performance cathode materials of LIBs.

## Data Availability Statement

The datasets generated for this study are available on request to the corresponding author.

## Author Contributions

SZ, XZ, and JL did the main experiment and wrote the manuscript. JL and YS were involved the discussion of the experiment, revised the manuscript, and made the research plan. XZ assisted in the material synthesis. SZ and YS also provided the financial support.

## Conflict of Interest

The authors declare that the research was conducted in the absence of any commercial or financial relationships that could be construed as a potential conflict of interest.

## References

[B1] Abou-Rjeily J. Bezza I. Laziz N. A. Autret-Lambert C. Sougrati M. T. Ghamouss F. (2019). High-rate cyclability and stability of LiMn_2_O_4_ cathode materials for lithium-ion batteries from low-cost natural β-MnO_2_. Energy Storage Mater. 26, 423–432. 10.1016/j.ensm.2019.11.015

[B2] Barker J. Saidi M. Y. Swoyer J. L. (2003). Electrochemical insertion properties of the novel lithium vanadium fluorophosphates. J. Electrochem. Soc. 150, A1394–A1398. 10.1149/1.1609998

[B3] El-Bana M. S. El Radaf I. M. Fouad S. S. Sakr G. B. (2017). Structural and optoelectrical properties of nanostructured LiNiO_2_ thin films grown by spray pyrolysis technique. J. Alloy. Compd. 705, 333–339. 10.1016/j.jallcom.2017.02.106

[B4] Goodenough J. B. Park K. S. (2013). The Li-ion rechargeable battery: a perspective. J. Am. Chem. Soc. 135, 1167–1176. 10.1021/ja3091438 23294028

[B5] Gover R. K. B. Burns P. Bryan A. Saidi M. Y. Swoyer J. L. Barker J. (2006). LiVPO_4_F: A new active material for safe lithium-ion batteries. Solid State Ion. 177, 2635–2638. 10.1016/j.ssi.2006.04.049

[B6] Hariprasad K. Naresh N. Nageswara Rao B. Venkateswarlu M. Satyanarayana N. (2016). Preparation of LiMn_2_O_4_ nanorods and nanoparticles for lithium-ion battery applications. Mater. Today Proc. 3, 4040–4045. 10.1016/j.matpr.2016.11.070

[B7] Hu G. Gan Z. Cao Y. Peng Z. Lu Y. Du K. (2019). Multi-level carbon co-modified LiVPO_4_F cathode material for lithium batteries. J. Alloy. Compd. 788, 1146–1153. 10.1016/j.jallcom.2019.02.323

[B8] Huang H. Faulkner T. Saidi M. Y. (2009). Lithium metal phosphates, power and automotive applications. J. Power Sources 189, 748–751. 10.1016/j.jpowsour.2008.08.024

[B9] Jiang S. (2018). Ultrafine Li_3_V_2_(PO_4_)_3_ crystals adhered to P-doped graphene sheets for electrochemical lithium storage. Solid State Ionics 326, 58–62. 10.1016/j.ssi.2018.09.005

[B10] Karami H. Taala F. (2011). Synthesis, characterization and application of Li_3_Fe_2_(PO_4_)_3_ nanoparticles as cathode of lithium-ion rechargeable batteries. J. Power Sources 196, 6400–6411. 10.1016/j.jpowsour.2011.03.079

[B11] Kim M. Kang B. (2017). Highly-pure triplite 3.9V-LiFeSO_4_F synthesized by a single-step solid-state process and its high electrochemical performance. Electrochim. Acta 228, 160–166. 10.1016/j.electacta.2017.01.073

[B12] Li R. Sun S. Liu J. Chen T. Dai C. Ding F. (2019). From rational construction to theoretical study: Li_3_V_2_(PO_4_)_3_ nanoplates with exposed {100} facets for achieving highly stable lithium storage. J. Power Sources 442:227231 10.1016/j.jpowsour.2019.227231

[B13] Li Y. Wang J. Yao J. Huang H. Du Z. Gu H. (2019). Enhanced cathode performance of LiFePO_4_/C composite by novel reaction of ethylene glycol with different carboxylic acids. Mater. Chem. Phys. 224, 293–300. 10.1016/j.matchemphys.2018.12.042

[B14] Lin J. Zeng C. Wang L. Pan Y. Lin X. Reddy R. C. K. (2020). Self-standing MOF-derived LiCoO_2_ nanopolyhedron on Au-coated copper foam as advanced 3D cathodes for lithium-ion batteries. Appl. Mater. Today 19:100565 10.1016/j.apmt.2020.100565

[B15] Nitta N. Wu F. Lee J. T. Yushin G. (2015). Li-ion battery materials: present and future. Mater. Today 18, 252–264. 10.1016/j.mattod.2014.10.040

[B16] Sui Y. Chen W. Tang S. Wu L. Wang B. Li H.. (2019a). Spray-drying synthesis of LiFeBO_3_/C hollow spheres with improved electrochemical and storage performances for Li-ion batteries. Front. Chem. 7:379. 10.3389/fchem.2019.00379 31192195 PMC6546830

[B17] Sui Y. Wu L. Hong W. Liu J. Zhang X. Li W. (2019b). Synthesis and electrochemical properties of spherically shaped LiVPO_4_F/C cathode material by a spray drying-roasting method. Rare Met. 10.1007/s12598-019-01340-0

[B18] Uddin M. Alaboina P. K. Cho S. (2017). Nanostructured cathode materials synthesis for lithium-ion batteries. Mater. Today Energy 5, 138–157. 10.1016/j.mtener.2017.06.008

[B19] Vásquez F. A. Calderón J. A. (2019). Vanadium doping of LiMnPO_4_ cathode material: correlation between changes in the material lattice and the enhancement of the electrochemical performance. Electrochim. Acta 325:134930 10.1016/j.electacta.2019.134930

[B20] Wu L. Zheng J. Wang L. Xiong X. Shao Y. Wang G.. (2019). PPy-encapsulated SnS_2_ nanosheets stabilized by defects on a TiO_2_ support as a durable anode material for lithium-ion batteries. Angew. Chem. Int. Edit. 58, 811–815. 10.1002/anie.201811784 30417513

[B21] Wu X. Meledina M. Tempel H. Kungl H. Mayer J. Eichel R. (2020). Morphology-controllable synthesis of LiCoPO_4_ and its influence on electrochemical performance for high-voltage lithium ion batteries. J. Power Sources 450:227726 10.1016/j.jpowsour.2020.227726

[B22] Zhang S. Gu Q. Tan S. Zhao L. (2019). Improved electrochemical properties of the Li_3_V_2_(PO_4_)_3_ cathode material synthesized from a V(III) precursor. J. Alloy. Compd. 802, 583–590. 10.1016/j.jallcom.2019.06.240

[B23] Zhao H. Wang J. Wang G. Liu S. Tan M. Liu X. (2017). Facile synthesis of orthorhombic LiMnO_2_ nanorods by in-situ carbothermal reduction: promising cathode material for Li ion batteries. Ceramics Int. 43, 10585–10589. 10.1016/j.ceramint.2017.04.158

[B24] Zheng J. Han Y. Sun D. Zhang B. Cairns E. J. (2017). In situ-formed LiVOPO_4_@V_2_O_5_ core-shell nanospheres as a cathode material for lithium-ion cells. Energy Storage Mater. 7, 48–55. 10.1016/j.ensm.2016.12.003

[B25] Zheng J. Han Y. Tang L. Zhang B. (2016). Investigation of phase structure change and electrochemical performance in LiVP_2_O_7_-Li_3_V_2_(PO_4_)_3_-LiVPO4F system. Electrochim. Acta 198, 195–202. 10.1016/j.electacta.2016.03.070

[B26] Zheng J. Yang Z. Dai A. Tang L. Wei H. Li Y. (2019). Boosting cell performance of LiNi_0.8_Co_0.15_Al_0.05_O_2_ via surface structure design. Small 15:1904854 10.1002/smll.201904854 31724336

